# Downregulation of SLC7A7 Triggers an Inflammatory Phenotype in Human Macrophages and Airway Epithelial Cells

**DOI:** 10.3389/fimmu.2018.00508

**Published:** 2018-03-19

**Authors:** Bianca Maria Rotoli, Amelia Barilli, Rossana Visigalli, Filippo Ingoglia, Marco Milioli, Maria Di Lascia, Benedetta Riccardi, Paola Puccini, Valeria Dall’Asta

**Affiliations:** ^1^Department of Medicine and Surgery (DiMeC), University of Parma, Parma, Italy; ^2^Preclinical Pharmacokinetics, Biochemistry and Metabolism Department, Chiesi Farmaceutici, Parma, Italy

**Keywords:** LPI, innate immunity, cytokine and chemokines, inflammation, arginine

## Abstract

Lysinuric protein intolerance (LPI) is a recessively inherited aminoaciduria caused by mutations of SLC7A7, the gene encoding y+LAT1 light chain of system y^+^L for cationic amino acid transport. The pathogenesis of LPI is still unknown. In this study, we have utilized a gene silencing approach in macrophages and airway epithelial cells to investigate whether complications affecting lung and immune system are directly ascribable to the lack of SLC7A7 or, rather, mediated by an abnormal accumulation of arginine in mutated cells. When SLC7A7/y+LAT1 was silenced in human THP-1 macrophages and A549 airway epithelial cells by means of short interference RNA (siRNA), a significant induction of the expression and release of the inflammatory mediators IL1β and TNFα was observed, no matter the intracellular arginine availability. This effect was mainly regulated at transcriptional level through the activation of NFκB signaling pathway. Moreover, since respiratory epithelial cells are the important sources of chemokines in response to pro-inflammatory stimuli, the effect of IL1β has been addressed on SLC7A7 silenced A549 cells. Results obtained indicated that the downregulation of SLC7A7/y+LAT1 markedly strengthened the stimulatory effect of the cytokine on CCL5/RANTES expression and release without affecting the levels of CXCL8/IL8. Consistently, also the conditioned medium of silenced THP-1 macrophages activated airway epithelial cells in terms of CCL5/RANTES expression due to the presence of elevated amount of proinflammatory cytokines. In conclusion, our results point to a novel thus far unknown function of SLC7A7/y+LAT1, that, under physiological conditions, besides transporting arginine, may act as a brake to restrain inflammation.

## Introduction

Lysinuric protein intolerance (LPI; MIM 222700) is a recessively inherited aminoaciduria caused by mutations of SLC7A7, the gene encoding y+LAT1 light chain of system y^+^L for cationic amino acid transport (1, 2). LPI is a severe multisystem disorder characterized by protein-rich food intolerance with secondary urea cycle disorders; symptoms are heterogeneous and include failure to thrive, recurrent vomiting, episodes of hyperammonemic coma after a protein-rich meal, hepatosplenomegaly, muscular hypotonia, osteoporosis, lung involvement, kidney failure, hematologic abnormalities, and immunological disorders with autoimmunity (3).

The physiopathology of LPI is thus far only partially elucidated. The classical hallmarks of the disease, i.e., leakage of cationic amino acids in the urine and normal-to-low plasma levels, are directly ascribable to a defective intestinal absorption of the amino acids, combined with an increased loss in the kidney due to SLC7A7/y+LAT1 mutation in the basolateral membrane of intestinal and renal epithelial cells (4–7); the resulting deficiency of urea cycle intermediates in blood leads to a functional impairment of the cycle that easily explains hyperammonemia and, in turn, acute symptoms after protein ingestion, such as nausea and vomiting (8). Conversely, a nutritional imbalance in LPI patients cannot give reason of the complex multiorgan manifestations of the disease that represent the major causes of morbidity and mortality.

As for lung complications, LPI patients frequently manifest with progressive pulmonary tissue changes typical of interstitial lung disease (ILD) (9), a collection of disorders characterized by inflammation and fibrosis in the lung. Traditionally, pulmonary fibrotic processes were considered a chronic inflammatory-driven response caused by the abnormal accumulation of inflammatory cells, such as alveolar macrophages, but current evidence suggests that the fibrotic response could also be driven by abnormally activated airway epithelial cells (10). In LPI, in particular, a well-known life-threatening complication of ILD is the progression to severe pulmonary alveolar proteinosis (PAP) with an excessive deposition of surfactant-like lipoproteinaceous material within alveoli, which often accounts for the fatal outcome due to respiratory failure (11). In addition, LPI associates with an increased incidence of hemophagocytic lymphohistiocytosis (HLH) (12, 13), a wide array of related life-threatening conditions in which uncontrolled and self-sustained activation of lymphocytes and macrophages leads to a severe hyper-inflammatory state. A variety of disease mechanisms can lead to HLH: while primary forms, also known as familial HLH, are caused by genetic defects that impair lymphocytes’ cytotoxic function, acquired HLH are secondary to infections, autoimmune and autoinflammatory diseases, malignancy or metabolic disorders such as LPI (14). In both cases, an excessive cytokine production (“cytokine storm”) occurs, due to the over-activation of macrophages and cytotoxic lymphocytes that infiltrate tissues, leading to multiorgan dysfunction and, ultimately, to an increased mortality (15). Moreover, clinical and biochemical findings consistent with systemic lupus-like syndromes have been also reported in LPI patients (3, 16, 17), suggesting a role for immune dysfunctions in the onset of the disease; systemic lupus erythematosus (SLE) is, indeed, a chronic and complex autoimmune condition that leads to widespread inflammation and tissue injury in multiple organs (18).

Although the pathogenetic mechanisms of LPI and its complications are still unclear, the involvement of the mononuclear phagocyte system is nowadays well recognized. Indeed, the case of an LPI patient has been reported, who underwent heart-lung transplantation for severe PAP-associated respiratory insufficiency, relapsed and died of respiratory failure after a period of clinical remission (19), suggesting that circulating cells, likely monocytes/macrophages, colonized the transplanted lung and reproduced the pathological condition. Moreover, experimental evidence obtained by our and other groups demonstrated that monocytes/macrophages typically express high levels of SLC7A7 (20) and that LPI macrophages display an impaired phagocytosis (21), as well as dysfunctions in toll-like receptor signaling (22).

In light of these findings, it has been speculated that immunological complications of LPI could be ascribable to alterations of bone marrow-derived monocytes that make them differentiating into dysfunctional macrophages. More precisely, since y+LAT1 operates as an efflux route for arginine (23), LPI defect is expected to cause an abnormal accumulation of the amino acid inside the cells (21, 23); hence, Sebastio et al. proposed that immune dysfunctions in LPI may be due to alterations of arginine intracellular metabolic routes in mononuclear cells, with an hyper-induction of the pro-inflammatory pathway of nitric oxide synthase (3). Although this hypothesis appears intriguing, we explore here the possibility that y+LAT1 protein is endowed with thus far unknown immunomodulatory functions and that SLC7A7 mutation, besides affecting arginine transport and intracellular availability, also directly modulates the inflammatory phenotype of macrophages and airway epithelial cells.

## Materials and Methods

### Cell Models and Cultures

THP-1 cells (ATCC^®^ TIB-202™) were cultured in arginine-free RPMI1640 medium (Biochrom AG, Germany) added with different concentrations of the amino acid (0.1, 1.0, and 10 mM); the medium was supplemented with 10% fetal bovine serum (FBS) and 1% penicillin/streptomycin and cells were routinely maintained in suspension under exponential growth at a density ranging from 2 × 10^5^ to 1 × 10^6^ cells/ml, with serial passages in culture flasks (Falcon, Steroglass, Italy). For the experiments, cells underwent gene silencing of SLC7A7/y+LAT1, as already described (23). Briefly, a suspension of cells (1.5 × 10^6^/ml) was diluted with an equal volume of serum free RPMI1640 containing HiPerFect Transfection Reagent (Qiagen^®^, Italy; 30 µl/ml) and 60 nM scrambled (AllStar Negative Control; Qiagen^®^; Cat.# SI03650318) or SLC7A7 (FlexiTube short interfering RNA; Qiagen^®^; Cat.# SI00058660) siRNA; after 4 h cell suspension was further diluted 1:3 with fresh, complete culture medium. After 24 h, cells were induced to differentiate by addition of phorbol-12-myristate-13-acetate (PMA; 80 nM) and maintained in culture for further 72 h. When caspase-1 inhibition was required, 10 µM z-WHED-FMK (R&D System, Space, Italy) was added 1 h before the addition of PMA. When indicated, the incubation medium of THP-1 cells was employed as conditioned medium (CM) for the treatment of A549 cells (see below).

Human pulmonary epithelial A549 cells (ATCC^®^ CCL-185™) were routinely cultured in Dulbecco’s Modified Eagle Medium (DMEM, high glucose) supplemented with sodium pyruvate (1 mM), 10% fetal bovine serum (FBS) and 1% Penicillin/Streptomycin. When gene silencing was required, cells were transfected according to the fast-forward transfection protocol provided by the manufacturer (Qiagen^®^): cells (1 × 10^5^/ml) were transfected by adding 0.2 volume of serum-free medium containing HiPerFect Transfection Reagent (9 µl/ml) and 100 nM scrambled or SLC7A6/y+LAT2 (FlexiTube short interfering RNA; Qiagen^®^; Cat.# SI00058681) siRNA; transfected cells were seeded as required by the experimental plan and maintained under growing conditions for 96 h.

### RT-qPolymerase Chain Reaction

1 µg of total RNA, isolated with GenElute™ Mammalian Total RNA Miniprep Kit (Sigma, Italy), was reverse transcribed and 40 ng of cDNA was amplified as described previously (20) with the following primers (5 pmol each): 5’ CCA GCA GTC GTC TTT GTC AC 3’ and 5’ CTC TGG GTT GGC ACA CAC TT 3’ for CCL5/RANTES; 5’ ACT GAG AGT GAT TGA GAG TGG AC 3’ and 5’ AAC CCT CTG CAC CCA GTT TTC 3’ for CXCL8/IL8; 5’ ACA GAC CTT CCA GGA GAA TG 3’ and 5’ GCA GTT CAG TGA TCG TAC AG 3’ for IL1B; and 5’ ATG AGC ACT GAA AGC ATG ATC C 3’ and 5’ GAG GGC TGA TTA GAG AGA GGT C 3’ for TNFA. The expression of SLC7A6/y+LAT2, SLC7A7/y+LAT1 and that of the housekeeping gene RPL15 (Ribosomal Protein Like 15) were monitored employing specific TaqMan^®^ Gene Expression Assays (Life Technologies Italia, Milano, Italy; Cat# Hs00187757_m1, Hs00909952_m1, and Hs03855120_g1, respectively), according to the manufacturer’s instructions. The expression of the gene of interest under each experimental condition was normalized to that of the housekeeping gene, according to the comparative CT method (ΔΔCT Method) (24), and shown as “fold increase” with respect to its levels in cells transfected with scrambled siRNA (= 1).

### l-Arginine Influx

Four distinct mechanisms, named systems y^+^, y^+^L, b^0,+^, and B^0,+^, account for arginine transport in mammalian tissues (see (25) for review); their contribution to total uptake can be discriminated on the basis of their differential sensitivity to inhibition by neutral amino acids in the absence or in the presence of sodium (25).

For the characterization of arginine transport in alveolar cells, A549 cells were cultured onto 96-well trays (Falcon). After two rapid washes in pre-warmed transport buffer [Earle’s Balanced Salt Solution, EBSS, containing (in mM) 117 NaCl, 1.8 CaCl_2_, 5.3 KCl, 0.9 NaH_2_PO_4_, 0.8 MgSO_4_, 5.5 glucose, 26 Tris/HCl, adjusted to pH 7.4], cells were incubated for 1 min in the same solution containing [^3^H]arginine (50 µM, 5 μCi/ml) in the absence or presence of 2 mM leucine or 2 mM leucine + lysine, so as to discriminate the contribution of systems y^+^L and y^+^. When sodium-independent transport was to be measured, a modified Na^+^-free EBSS (NMG-EBSS) was employed, with NaCl and NaH_2_PO_4_ replaced by *N*-methyl-d-glucamine and choline salts, respectively. The experiment was terminated by two rapid washes (<10 s) in ice-cold 300 mM urea. The intracellular pool of cell monolayers was extracted in ethanol, and radioactivity in cell extracts was determined with Wallac Microbeta Trilux liquid scintillation spectrometer (PerkinElmer, Italy). l-Arginine uptake was normalized for protein content, determined directly in each well by using a modified Lowry procedure (26) and expressed as pmol/mg of protein/min. Since an excess of neutral amino acids inhibits system y^+^L activity while an excess of leucine and lysine hinders all saturable components, the contribution of systems y^+^L was calculated as the difference between total uptake and the uptake obtained in the presence of 2 mM leucine, and system y^+^ activity was calculated as the difference between the uptake measured in the presence of 2 mM leucine and in presence of 2 mM leucine + lysine.

### Determination of Arginine Intracellular Content

For the determination of the intracellular content of arginine, cells were rapidly washed with PBS and the intracellular pool, extracted with a 10 min-incubation in acetonitrile/water (1:1) at 4°C, was analyzed by HPLC-ESI-MS/MS as previously described (27), with minor modifications. Briefly, HPLC separation was carried out on Ascentis Express HILIC column (Supelco, Bellefonte, PA, USA) at 35°C. Analytes (injection volume corresponding to 10 µl) were chromatographically separated under optimized gradient; mass spectrometric analyses were carried out using an AB SCIEX 4500 Q-TRAP (AB Sciex, Foster City, CA, USA) with a Turbo Ion Spray probe in positive mode. The monitored transitions for l-arginine and ^15^N_2_-Arginine (used as internal standard) were 174.739 *m/z*→70.031 *m/z* and 176.739 *m/z*→70.031 *m/z*, respectively. Protein content in each condition was determined using a modified Lowry procedure (28) and l-arginine content was expressed as nmol/mg of protein.

### Determination of Cytokine and Chemokine Production

Cell culture supernatants were collected under the indicated experimental conditions, centrifuged for 10 min at 250 g and kept at −20°C for the measurements of cytokines and chemokines. IL1β, IL8, RANTES, and TNFα in the extracellular medium were, then, quantified using Quantikine ELISA kit (R&D System), Human IL8 Picokine ELISA Kit (Vinci Biochem), Human RANTES ELISA Kit (MyBioSource), and Human TNFα ELISA Kit (Immulological Sciences), respectively. All measurements were made following manufacturers’ instructions.

### Determination of Caspase-1 Activity

The activation of caspase-1 was monitored in THP-1 cell lysates by employing the commercially available Caspase-1/ICE Colorimetric Assay Kit by R&D System, according to the manufacturer’s instruction.

### Western Blot Analysis

The expression of y+LAT2 and y+LAT1 proteins was determined in whole protein lysates obtained as already described (23), while the activation of NF-κB pathway was monitored in nuclear extracts isolated with the Nuclear Extract Kit (Active Motif, Vinci-Biochem, Italy); Western Blot analyses were, then, performed as described previously (23). Briefly, 20 µg of proteins were separated by SDS-PAGE (10% acrylamide) and electrophoretically transferred to PVDF membranes (Immobilione-P membrane; Millipore, Italy). Membranes were incubated for 1 h at RT in Tris-buffered saline solution (TBS; 50 mM Tris-HCl pH 7.5, 150 mM NaCl) containing 5% non-fat dried milk, then incubated overnight at 4°C in TBS added with 3% non-fat dried milk and anti-NF-κB p65 (1:1000; Santa Cruz Biotechnology, Italy), anti-y+LAT2, or anti-y+LAT1 (1:500; Thermo Fisher Scientific, Italy) antibodies. PARP or α-actin, detected with polyclonal antibodies (1:1000; Cell Signaling Technology, EuroClone, Italy or Sigma Aldrich, respectively), was employed as internal standard. Immunoreactivity was visualized with enhanced chemiluminescence (Millipore).

### Statistics

Statistical analysis has been performed with Prism^®^5.0 GraphPad software. Gaussian distribution has been preliminary verified; then parametric tests have been employed to calculate statistical significance, as specified for each figure. Differences were considered significant when *p* < 0.05.

### Materials

Endotoxin-free fetal bovine serum (FBS) was purchased from EuroClone (Italy); l-[2,3,4-^3^H]Arginine monohydrochloride (43 Ci/mmol) was obtained from Perkin-Elmer (Italy) and GM-CSF from Vinci-Biochem (Italy). Unless otherwise stated, Sigma-Aldrich (Italy) was the source of all the other chemicals.

## Results

### SLC7A7 Gene Silencing Induces a Pro-Inflammatory Pathway in THP-1 Macrophages

**Figure 1:**
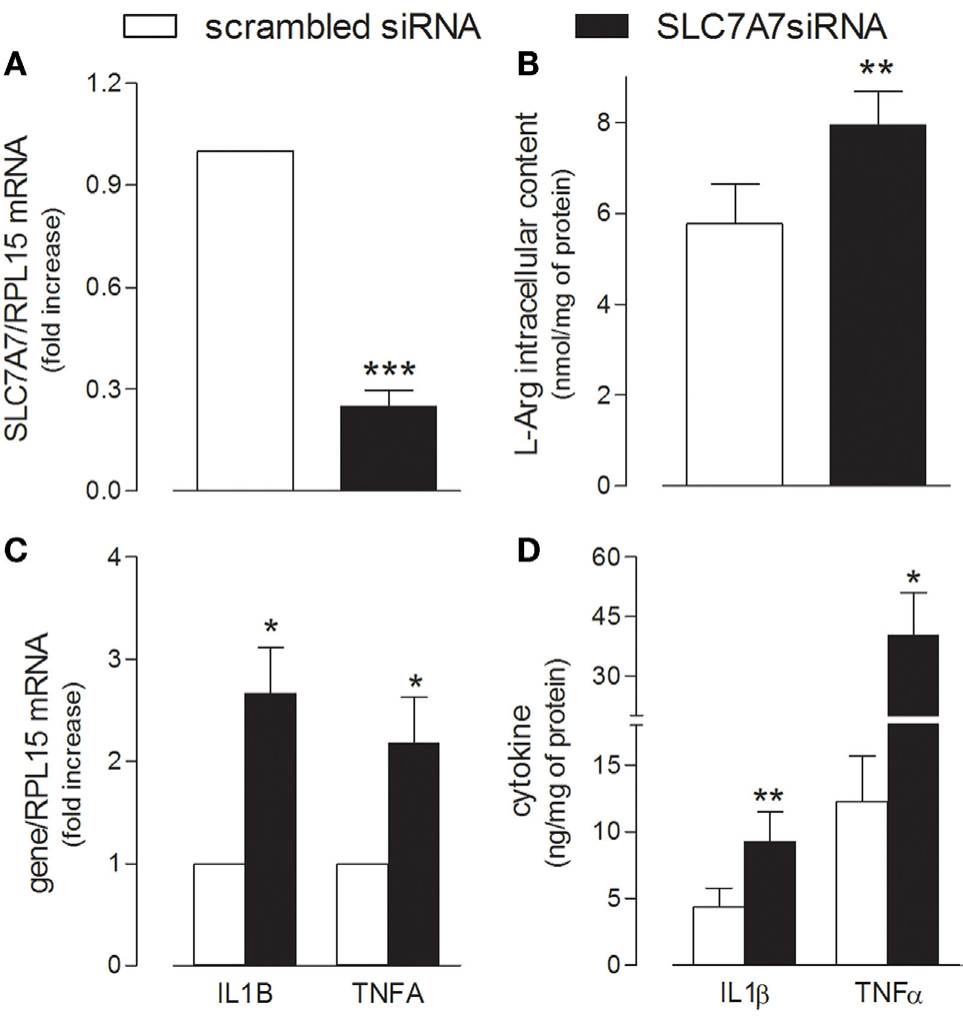
THP-1 cells, routinely grown in the presence of 1 mM extracellular arginine, were transfected with scrambled or SLC7A7 short interference RNA (siRNA) in the presence of 80 nM phorbol-12-myristate-13-acetate (PMA), as described in Section “Materials and Methods”; after 96 h, cells were analyzed for the expression of SLC7A7 mRNA with RT-qpolymerase chain reaction (RT-qPCR) **(A)** and for intracellular arginine content by means of HPLC/ESI-MS-MS **(B)**. In the same cells, the production and release of IL1β and TNFα were monitored with RT-qPCR **(C)** and ELISA assay **(D)**, respectively. Data are mean ± SEM of four different determinations, each performed in duplicate. *p < 0.05, **p < 0.01, ***p < 0.001 vs. scrambled siRNA with One sample *t*-test **(A,C)** or Student’s *t*-test for paired samples **(B,D)**.

In order to reproduce *in vitro* the genetic defect of LPI macrophages, SLC7A7/y+LAT1 was silenced by means of short interference RNA (siRNA) in PMA-differentiated human THP-1 cells (Figure 1). A significant decrease of mRNA and protein expression was observed in THP-1 cells after 72 h gene silencing (Figure 1A), as previously reported (23). Under the same experimental conditions, arginine intracellular content was slightly, but significantly higher in silenced (SLC7A7 siRNA) than in control (scrambled siRNA) cells (Figure 1B). Interestingly, the decrease of SLC7A7 expression was paralleled by the simultaneous induction of a pro-inflammatory phenotype in treated macrophages: a significant increase of the mRNAs coding for interleukin 1β (IL1β) and tumor necrosis factor-alpha (TNFα) was, indeed, observed upon gene silencing (Figure 1C), along with an increased release of the same cytokines in the incubation medium (Figure 1D).

**Figure 2:**
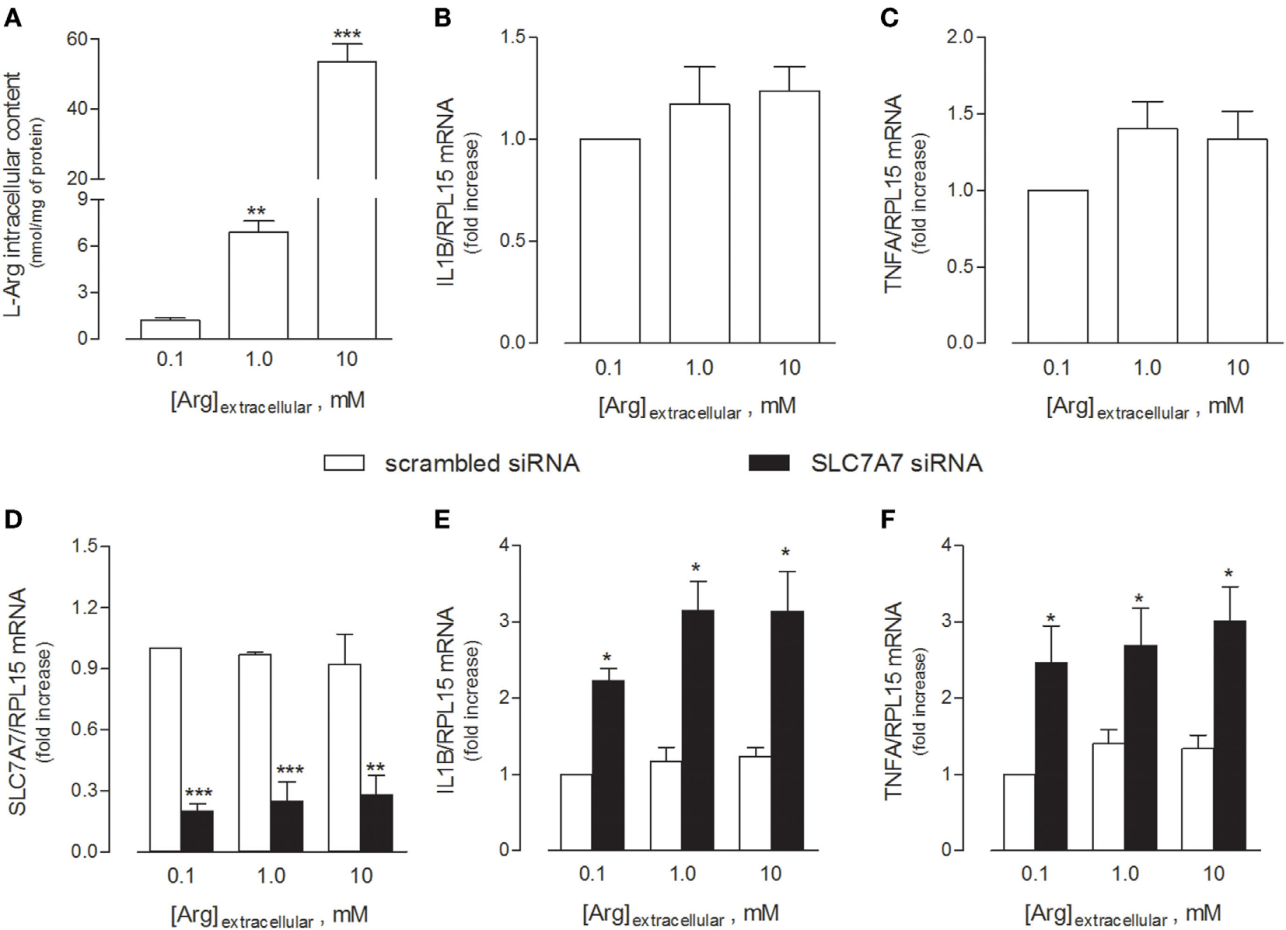
THP-1 cells were grown in the presence of the indicated concentrations of extracellular arginine. **(A–C)** Cells were differentiated to macrophages by exposure to 80 nM phorbol-12-myristate-13-acetate (PMA) for 96 h, then arginine content was measured by means of HPLC/ESI-MS-MS **(A)**, while the expression of IL1β **(B)** and TNFα **(C)** mRNAs was monitored with RT-qpolymerase chain reaction (RT-qPCR). Data are mean ± SEM of five independent determinations. *** *p* < 0.001 vs. [Arg]_extracellular_ 0.1 mM. **(D–F)** Cells were transfected with scrambled or SLC7A7 short interference RNA (siRNA) along the PMA-induced differentiation, as described in Section “Materials and Methods,” and analyzed for the expression of SLC7A7 **(D)**, IL1β **(E)**, and TNFα **(F)** mRNAs with RT-qPCR. Data are mean ± SEM of four different determinations, each performed in duplicate. *p < 0.05, **p < 0.01, ***p < 0.001 vs. scrambled siRNA with one-way ANOVA followed by Bonferroni *post hoc* test.

In light of these findings, we next explored whether the acquisition of an inflammatory behavior by macrophages upon SLC7A7 silencing was directly caused by the lack of y+LAT1 protein or, rather, mediated by intracellular arginine. To address this issue, THP-1 cells were cultured in the presence of different concentrations (0.1, 1, and 10 mM) of extracellular arginine, so as to modulate the intracellular amount of the amino acid (Figure 2). As shown in Figure 2A, arginine progressively accumulated inside the cells as its concentrations in the extracellular medium increased; however, no change in the expression of either IL1B (Figure 2B) or TNFA (Figure 2C) genes was observed. Moreover, SLC7A7 silencing at any concentration of arginine (Figure 2D) caused a significant increase of the mRNAs coding for IL1β (Figure 2E) and TNFα (Figure 2F), no matter the amount of the amino acid; the observed induction was comparable under the different experimental conditions despite the big differences in intracellular arginine content. These findings demonstrate that the acquired inflammatory behavior in SLC7A7 silenced cells is independent from arginine content.

**Figure 3:**
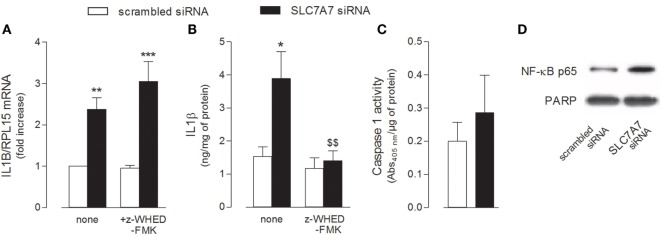
THP-1 cells, routinely grown in the presence of 1 mM extracellular arginine, were transfected for 96 h with scrambled or SLC7A7 short interference RNA (siRNA) during PMA-induced differentiation, as described in Section “Materials and Methods”; when indicated, gene silencing was performed in the absence (none) or in the presence of caspase-1 specific inhibitor (z-WHED-FMK). **(A–C)** The expression of IL1B mRNA was determined with RT-RT-qPCR **(A)**, IL1β release was measured with ELISA assay **(B)**, and the activity of caspase-1 was determined as described in Section “Materials and Methods” **(C)**. Data are mean ± SEM of three different determinations, each performed in duplicate. *p < 0.05, **p < 0.01, ***p < 0.001 vs. scrambled siRNA; ^$$^*p* < 0.01 vs. SLC7A7 siRNA with one-way ANOVA followed by Bonferroni *post hoc* test. **(D)** The amount of NFκB-p65 in the nuclear fraction was monitored with Western Blot analysis. The experiment was repeated twice with comparative results; a representative blot is shown.

The release of IL1β has been described as a two-step process involving (1) the NFκB-dependent transcription of the gene for the synthesis of the inactive 31-kDa pro-IL1β and (2) the cleavage of pro-IL1β into the biologically active 17-kDa IL1β protein through the inflammasome-dependent activation of caspase-1 (29). Hence, we evaluated the activity of both NF-κB and caspase-1 under our experimental conditions, so as to define the mechanism responsible for the release of IL1β observed in THP-1 cells upon SLC7A7 silencing. As shown in Figure 3, the inhibition of caspase-1 by Z-WEHD-FMK during the transfection had no effect on IL1B mRNA expression (Figure 3A), while it completely prevented the increase of cytokine secretion (Figure 3B); however, the activity of caspase-1 was comparable in THP-1 cells transfected with scrambled or SLC7A7 siRNA (Figure 3C), suggesting that gene silencing had no or, at most, slight effect on the induction of enzyme activity. Conversely, a higher translocation of the NF-κB subunit p65 inside the nucleus was observed upon gene silencing (Figure 3D), indicating that the induction of IL1β secretion due to SLC7A7 downregulation in macrophages is regulated at transcriptional level through the activation of NF-κB-dependent mechanisms.

### SLC7A7 Gene Silencing Strengthens IL1β-Driven Inflammatory Phenotype in A549 Cells

Given the frequent involvement of airways in LPI complications, we next addressed the impact of SLC7A7/y+LAT1 defect on the phenotype of an *in vitro* model of pulmonary cells, i.e., A549 human alveolar epithelial cells.

**Figure 4:**
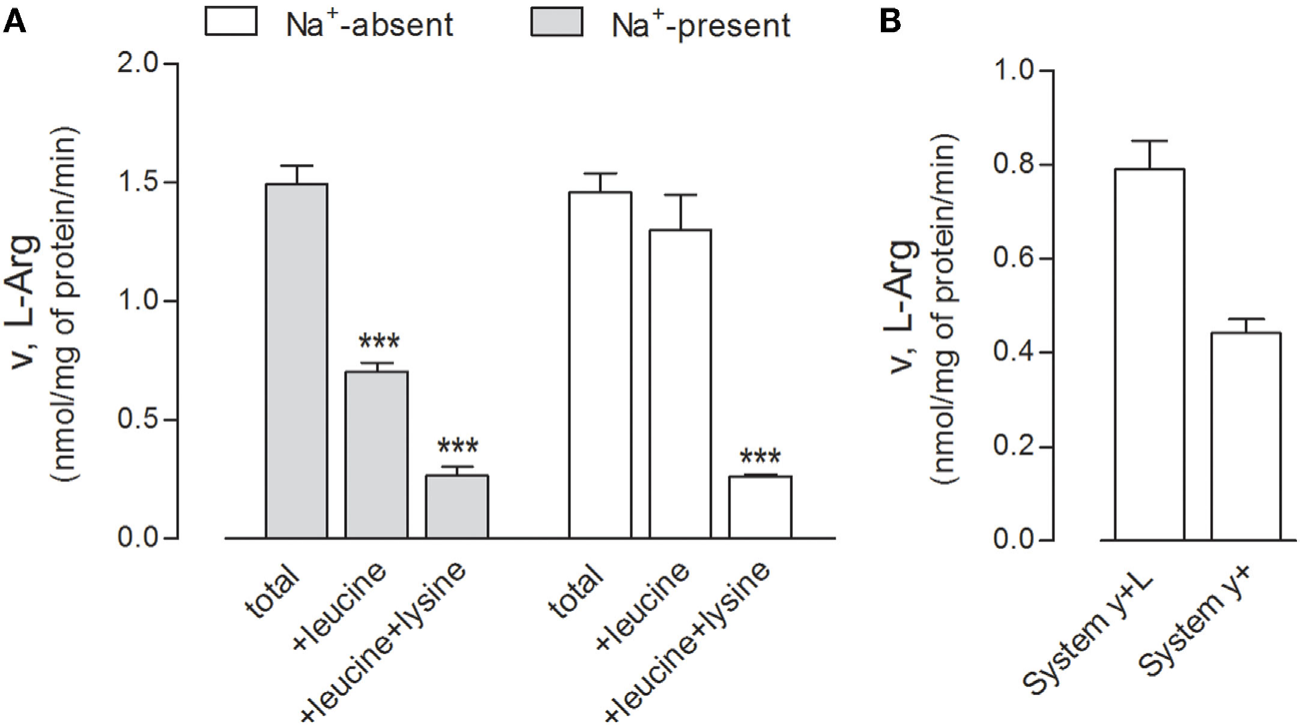
**(A)** A549 cells were washed in Earle’s balanced salt solution (EBSS) (Na^+^-present) or Na^+^-free NMG-EBSS (Na^+^-absent), as indicated; arginine uptake was then assayed with 1-min incubation in the same solution supplemented with l-[^3^H]arginine (0.05 mM; 3 μCi/ml) in the absence (total) or in the presence of 2 mM leucine or 2 mM leucine + lysine. Data are mean ± SD of five independent determinations in a representative experiment, which, repeated twice, gave comparable results. ***p < 0.001 vs. total uptake with one-way ANOVA followed by Bonferroni *post hoc* test. **(B)** Data shown in Panel **(A)** were employed to calculate the relative contribution of systems y^+^L and y^+^ to total arginine uptake (see Materials and Methods and Results).

First, we performed a preliminary characterization of the transport routes for arginine in these cells, since the transporters involved in the transmembrane fluxes of the amino acid have not been described thus far in human alveolar models. The relative contribution of each transport component has been identified employing a strategy already used in other cell models (21, 30); results are shown in Figure 4. Arginine transport was comparable in the absence and in the presence of sodium (Figure 4A), thus excluding a significant contribution of sodium-dependent transport systems, such as B^0,+^. The addition of leucine inhibited arginine influx by more than 50% only in the presence of sodium, demonstrating the operation of system y^+^L and excluding a significant contribution of the sodium-independent system b^0,+^. In the presence of sodium, the addition of 2 mM lysine to leucine produced a further decrease of transport, thus indicating that also system y^+^ contributes to arginine uptake. Thus, in A549 cells, both systems y^+^ and y^+^L account for arginine transport, with the latter representing the predominant route (Figure 4B).

**Figure 5:**
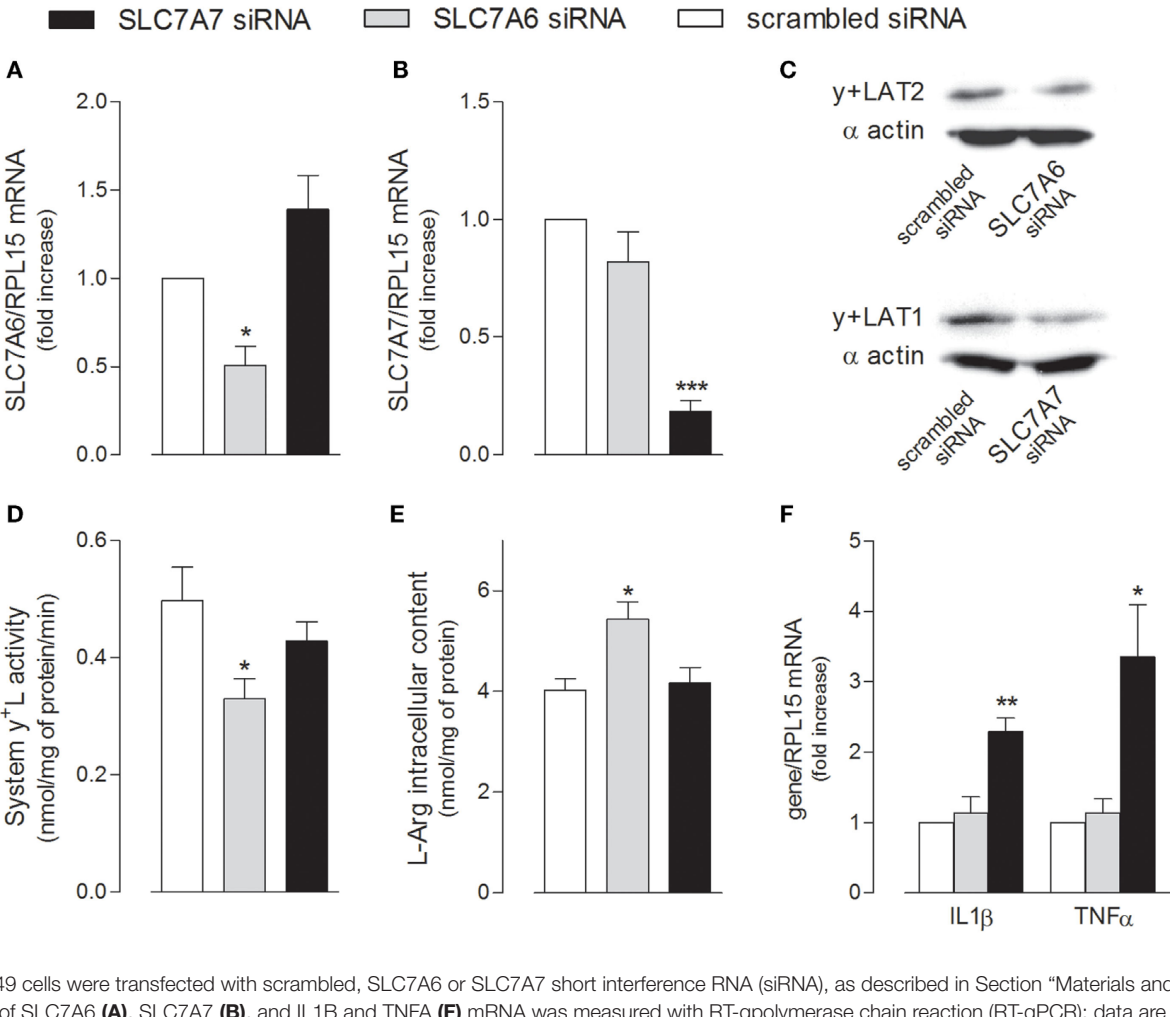
A549 cells were transfected with scrambled, SLC7A6 or SLC7A7 short interference RNA (siRNA), as described in Section “Materials and Methods.” The expression of SLC7A6 **(A)**, SLC7A7 **(B)**, and IL1B and TNFA **(F)** mRNA was measured with RT-qpolymerase chain reaction (RT-qPCR); data are mean ± SEM of three independent determinations, each performed in duplicate. Protein levels of y+LAT2 and y+LAT1 were determined with Western Blot analysis **(C)**; a representative blot is shown. The transport activity of System y^+^L **(D)** and arginine intracellular content **(E)** were also measured, as described in Section “Materials and Methods”; data are mean ± SEM of four independent experiments, each performed in quadruplicate. *p < 0.05, **p < 0.01, ***p < 0.001 vs. scrambled siRNA with one-way ANOVA followed by Bonferroni *post hoc* test.

The relative contribution of the two alternative light subunits SLC7A6/y+LAT2 and SLC7A7/y+LAT1 to the activity of system y+L transporters was then investigated by selectively silencing one or the other gene (Figure 5). Data obtained indicate that the expression of both transporters was efficiently and specifically reduced by siRNAs at both mRNA (Figures 5A,B) and protein level (Figure 5C); however, only the downregulation of SLC7A6 was able to slightly but significantly reduce arginine transport through system y+L (Figure 5D) and, in turn, to increase the intracellular content of the amino acid (Figure 5E). We can conclude that arginine availability in A549 cells is mainly regulated by SLC7A6/y+LAT2 activity. On the other hand, similarly to what observed in THP-1 macrophages (see Figure 1), the specific silencing of SLC7A7/y+LAT1 in A549 cells significantly induced the expression of IL1B and TNFA (Figure 5F), indicating that this protein modulates the inflammatory phenotype also of airway epithelial cells, with an effect that, even in this model, does not depend upon arginine content.

**Figure 6:**
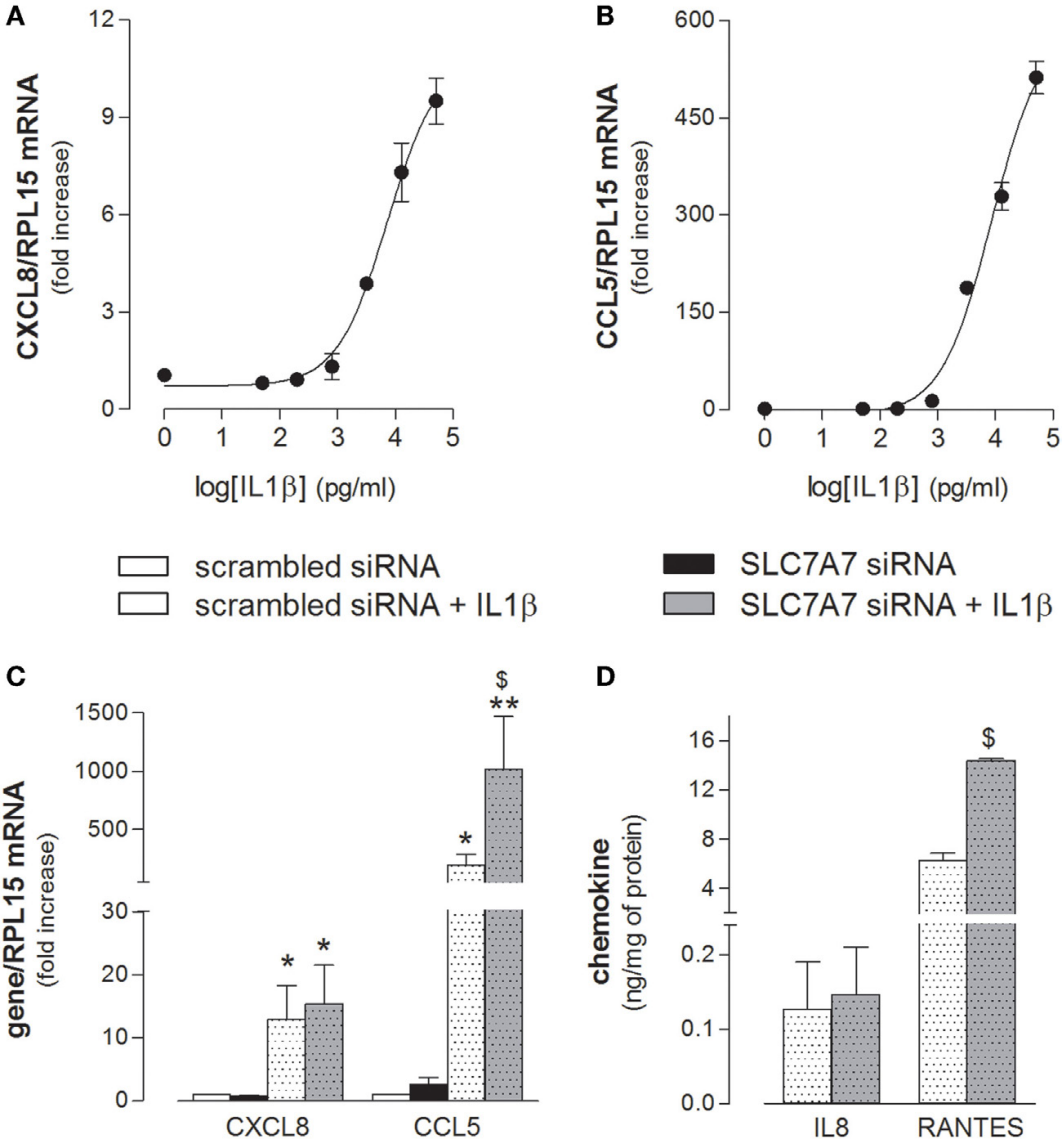
**(A,B)** A549 cells were incubated in the presence of the indicated concentrations of IL1β; after 24 h, the expression of CXCL8/IL8 and CCL5/RANTES was monitored with RT-qpolymerase chain reaction (RT-qPCR). Representative experiments are shown. **(C,D)** A549 cells were transfected with scrambled or SLC7A7 short interference RNA (siRNA) for 96 h (see Materials and Methods); where indicated, IL1β (10 ng/ml) was added to the incubation medium for the last 24 h; at the end, the expression and release of the indicated chemokines was monitored at mRNA **(C)** and protein **(D)** level with RT-qPCR and ELISA, respectively. Data are mean ± SEM of three independent determinations, each performed in duplicate. *p < 0.05, **p < 0.01 vs. scrambled siRNA; ^$^*p* < 0.05 vs. scrambled siRNA + IL1β with one-way ANOVA followed by Bonferroni *post hoc* test.

Next, we addressed whether SLC7A7/y+LAT1 was able to modulate A549 activation also in response to inflammatory stimuli. As shown in Figure 6, the mRNAs coding for CXCL8/IL8 and CCL5/RANTES chemokines was induced in a dose-dependent manner by exposure to IL1β (Figures 6A,B), with an EC[50] roughly corresponding to 7 and 9 ng/ml for IL8 and RANTES, respectively. Interestingly, the downregulation of SLC7A7 markedly strengthened the stimulatory effect of 10 ng/ml IL1β on the expression and release of CCL5/RANTES by airway cells, while the levels of CXCL8/IL8 were induced to the same extent in scrambled and gene silenced cells (Figures 6C,D).

**Figure 7:**
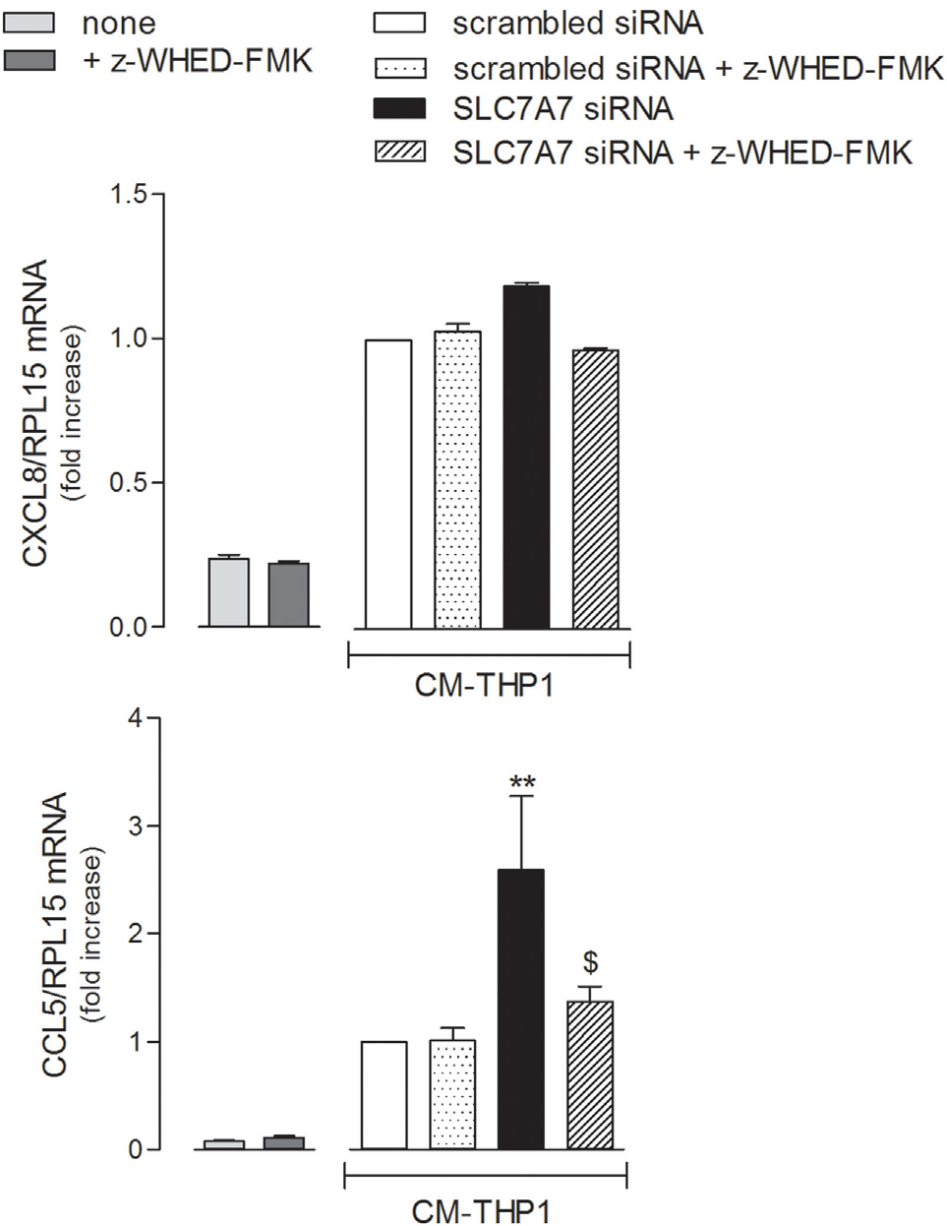
A549 were incubated with fresh medium (left panels) in the presence of caspase-1 inhibitor (+ z-WHED-FMK); in parallel, another set of A549 cells (right panels) was incubated with conditioned medium (CM) of THP-1 cells (CM-THP-1) silenced for 96 h with scrambled or SLC7A7 short interference RNA (siRNA), either in the absence or in the presence of caspase-1 inhibitor. After 24 h, the expression of CXCL8/IL8 and CCL5/RANTES was monitored with RT-qpolymerase chain reaction (RT-qPCR). Data are mean ± SEM of three independent determinations, each performed in duplicate. **p < 0.01 vs. scrambled siRNA CM-THP-1; ^$^*p* < 0.05 vs. SLC7A7 siRNA CM-THP-1 with one-way ANOVA followed by Bonferroni *post hoc* test.

In light of all the results obtained, we finally verified whether the inflammatory phenotype of airway epithelial cells may be modulated by SLC7A7/y+LAT1 silencing in macrophages. To this aim, A549 cells were treated with the medium obtained by incubating THP-1 with either scrambled or SLC7A7 siRNA, both in the absence and in the presence of caspase-1 inhibitor z-WHED-FMK (CM). As shown in Figure 7, the medium obtained upon SLC7A7 silencing caused a marked increase of CCL5/RANTES, but not of CXCL8/IL8 expression in A549 cells; this induction was significantly prevented when epithelial cells were treated with CM obtained by silencing SLC7A7 in the presence of caspase-1 inhibitor. This latter had *per se*, no effect on chemokine expression when added to A549 cells in fresh, not CM (left panels).

## Discussion

The most credited pathogenetic model of LPI to date identifies key injurious stimuli in an abnormal intracellular entrapping of arginine due to the absence of a functional 4F2hc/y+LAT1 complex and, in turn, in an unbalanced metabolism of the amino acid through pathways that differ among cell types (3); in particular, an overproduction of nitric oxide by nitric oxide synthase 2 (NOS2) in macrophages is supposed to be central to the development of immune dysfunctions (31). However, experimental evidence confirming the specific role of the amino acid and its metabolites in the onset of pulmonary and immunological disorders in LPI are, thus far, missing.

Aim of the present contribution was, then, to explore the hypothesis of a new, thus far unknown role for SLC7A7/y+LAT1, able to explain LPI complications. In this context, we demonstrate here for the first time that SLC7A7 gene silencing *in vitro* causes the acquisition of an inflammatory phenotype in both human macrophages and airway epithelial cells and that this effect does not depend on intracellular arginine content.

Indeed, the incubation of THP-1 cells at increasing concentrations of arginine actually raised the intracellular content of the amino acid, without triggering either IL1β or TNFα production; conversely, the downregulation of y+LAT1 was sufficient *per se* to induce the expression of both proinflammatory cytokines, no matter arginine availability (Figure 2). Similarly, in A549 cells, SLC7A7 silencing significantly increased cytokines production without affecting intracellular arginine content (Figure 5); conversely, the inhibition of SLC7A6/y+LAT2, which represents the major route for arginine in A549 cells, has no effect on cytokine release, despite it determine an increase of the amino acid inside the cells (Figures 4 and 5). These results point to a still unknown immunomodulatory function of y+LAT1 transporter, which does not depend on arginine availability.

IL1β production is a multistep process involving the NF-κB-dependent transcription of the gene and the next cleavage of pro-interleukin by caspase-1 in inflammasome (29). In our hands, the activity of both NF-κB and caspase-1 was detectable even in control conditions, i.e., in scrambled silenced cells, hence explaining the basal release of inflammatory cytokines; since phorbol esters are known to actually activate inflammasome in THP-1 cells (32), this finding is likely ascribable to phorbol-12-myristate-13-acetate (PMA) employed for cell differentiation. Anyway, when SLC7A7/y+LAT1 was silenced in macrophages, a more prominent nuclear translocation of p65 occurred, while the activity of caspase-1 was not altered; although the issue deserves to be better addressed, these results clearly ascribe the increase of IL1β secretion in silenced cells to an induction of NF-κB transcriptional activity, rather than to an hyper-activation of inflammasome. To this concern, the concomitant induction of TNFα mRNA reinforces the hypothesis of a key role for NF-κB in the effects observed upon SLC7A7 downregulation, since the same factor is notoriously responsible for the transcriptional regulation of this cytokine (33).

According to the results obtained, we speculate that y+LAT1 protein, under physiological conditions, acts as a brake to restrain inflammation, possibly through the repression of molecular pathways regulating NF-κB signaling; then, defects of y+LAT1 protein in LPI macrophages could explain, at least in part, the mechanisms underlying immunological and hematological complications of the disease. In this context, the clinical features resembling hemophagocytic lymphohistiocytosis (HLH) in LPI could be, hence, ascribed to a spontaneous and persistent activation of macrophages consequent to the lack of a functional SLC7A7/y+LAT1 in these cells. Consistently, an alternative mechanistic pathway for HLH immunopathology has been, indeed, recently proposed, in which recurrent fever and severe systemic inflammation are supposed to be the consequence of genetic defects primary impairing macrophages activation; in particular, gain-of-function mutations in inflammasome genes of macrophages and dendritic cells are shown to sustain HLH development, bypassing the requirement of cytotoxic lymphocytes (14).

To the same extent, an increased production of cytokines by macrophages and epithelial cells upon SLC7A7 silencing could give reason of pulmonary complications in LPI: on the one hand, the spontaneous production of inflammatory cytokines by y+LAT1 defective epithelial cells may stimulate them in an autocrine activation; on the other hand, SLC7A7/y+LAT1 defect in both cell types could exacerbates lung inflammation in LPI through a macrophage-epithelial crosstalk. Pro-inflammatory cytokines, besides viruses, microbes, allergens, and other environmental factors, are, indeed, known to be key modulators of immune and inflammatory reactions in the lungs, through the activation of respiratory epithelial cells (34). Activated epithelium is an important source of both CC and CXC chemokines, such as CXCL8/IL8 and CCL5/RANTES: IL8 acts as a key mediator in neutrophil-mediated acute inflammation (35), while RANTES is a potent chemoattractant for blood monocytes, T cells, and eosinophils (36). As expected, both proteins were upregulated in A549 cells by IL1β (Figure 6), the most potent inducer of chemokines in the respiratory epithelium (37). However, despite the increased expression of pro-inflammatory cytokines in SLC7A7 silenced A549 cells (see Figure 5), no significant change in chemokine expression was observed (see Figure 6), excluding a major contribution of autocrine mechanisms in the activation of airway epithelium in LPI. Consistently, clinical evidences sustain a central role of circulating immune cells in the development of lung pathology (19). Interestingly, the incubation of alveolar cells with CM of silenced THP-1 caused a slight but significant induction of CCL5/RANTES that was mainly imputable to the release of IL1β by macrophages upon SLC7A7 downregulation (Figure 7). Under the same condition, the expression of CXCL8/IL8 remained unaltered; it is important, however, to point out that IL1β in CM at most corresponds to 150–200 pg/ml, which is far below the minimum effective concentration of the cytokine on IL8 expression (see Figure 6, for comparison).

The stimulatory effect of IL1β on CCL5/RANTES production and release by A549 cells was reinforced by SLC7A7 silencing (Figure 6), suggesting that y+LAT1 defective epithelial cells in LPI airways could be more responsive to inflammatory cytokines than healthy cells. Interestingly, increased levels of RANTES have been detected in serum (38) and skin lesions (39) of SLE patients, pointing to a role for this chemokine in the inflammatory processes observed in lupus and LPI-related lupus-like syndromes.

**Figure 8:**
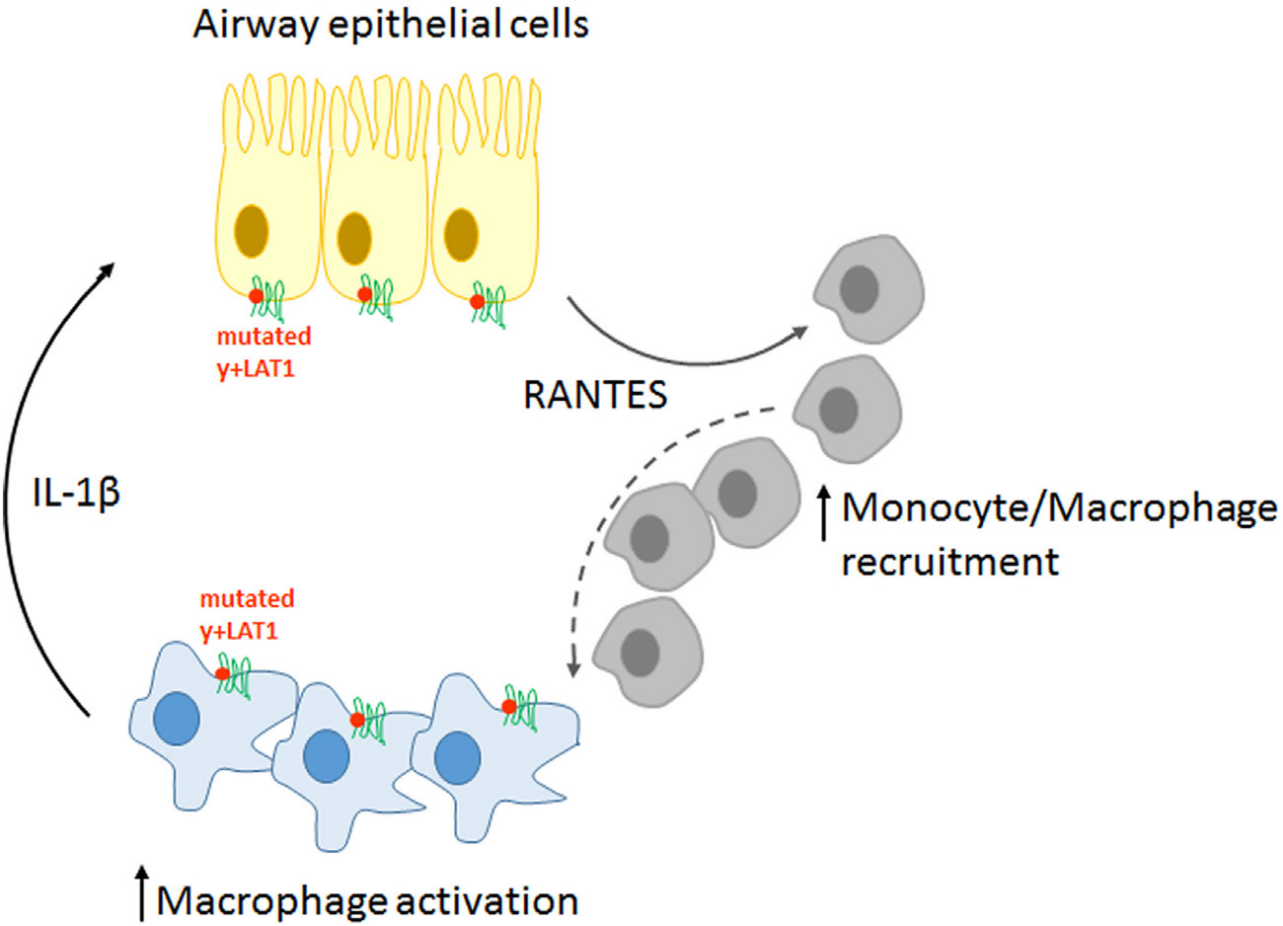
Schematic representation of the pathogenic model proposed for pulmonary complications in LPI.

Overall, our findings sustain a pathogenic model for LPI pulmonary complications, according to which immune and airway epithelial cells would be engaged in a positive feedback circuit responsible for explosive inflammatory responses (Figure 8). More precisely, pro-inflammatory cytokines spontaneously secreted by y+LAT1-defective monocytes and epithelial cells are expected to hyper-stimulate RANTES production by y+LAT1-defective airway epithelial cells that, in turn, would continuously recruit circulating monocytes to the airways. Consistently, macrophage-epithelial communications in the lung are emerging as central to the regulation of alveolar physiological homeostasis as well as of alveolar host-pathogen interactions (40).

This kind of mechanisms, which are notoriously central to fibrogenic responses in the lung (41, 42), in the liver (43), as well in the kidney (44) could give reason of LPI-associated fibrosis and would identify LPI as an auto-inflammatory disease, hence laying the bases for the development of alternative pharmacological approaches based on the use of anti-cytokines drugs.

## Author Contributions

BMR and VD designed the experimental plan. FI, AB, and RV performed *in vitro* experiments; MM and MDL performed HPLC-tandem mass spectrometry analysis; PP, BR, and VD analyzed the results. BMR and VD wrote the paper. All authors read and approved the final manuscript.

## Conflict of Interest Statement

The authors declare that the research was conducted in the absence of any commercial or financial relationships that could be construed as a potential conflict of interest.

## Abbreviations

EBSS, Earle’s balanced salt solution; PMA, phorbol-12-myristate-13-acetate; LPI, lysinuric protein intolerance; PAP, pulmonary alveolar proteinosis.
