# Supplementary material for: Downregulation of SLC7A7 Triggers an Inflammatory Phenotype in Human Macrophages and Airway Epithelial Cells
Source: Front Immunol. 2018 Mar 19;9:508. doi: 10.3389/fimmu.2018.00508 (PMC5868322; doi:10.3389/fimmu.2018.00508)
Supplement: Supplementary file 1 [file presentation_1.PDF]

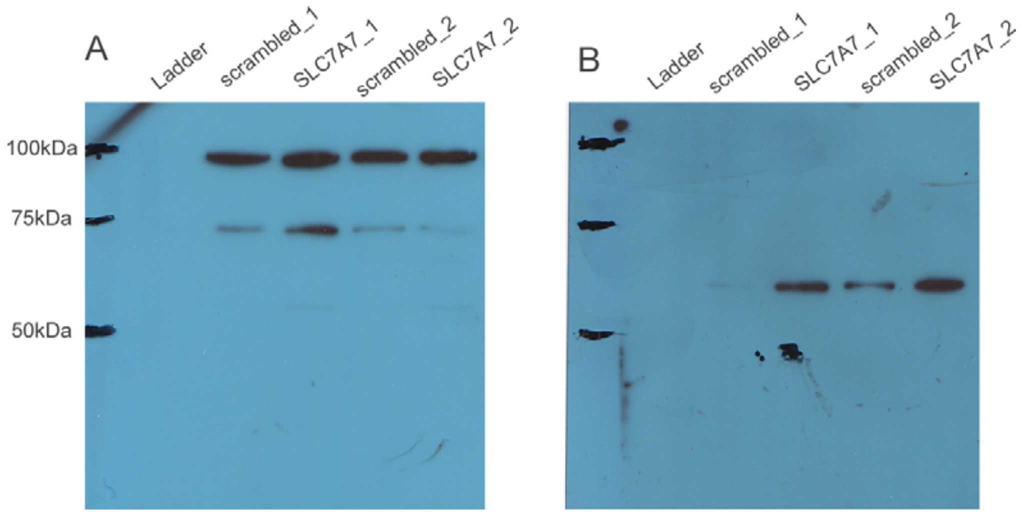

Figure S1. Blots for Figure 3D. Here above we show the original images of blots obtained for the analysis of NF-kB pathway in THP-1 cells upon SLC7A7 silencing; two independent experiments were performed (samples in Figure 3D are *scrambled_2* and *siRNA_2*).

Panel A: PARP, detected with a rabbit polyclonal antibody (1:1000; Cell Signaling Technology, EuroClone, Italy) and employed as internal standard; MW= 89 kDa. *Panel B*: NF-κB p65 obtained by employing a rabbit polyclonal antibody (1:1000, Santa Cruz Biotechnology, Italy); MW=65 kDa.

Lanes:

- ✓ *Ladder* is the marker for protein dimension (same sizes in A and B)
- ✓ *scrambled_1* and *scrambled_2* are lysates from cells transfected with scrambled siRNA in experiments 1 and 2, respectively;
- ✓ *SLC7A7_1* and *SLC7A7_2* are lysates from cells transfected with SLC7A7 siRNA in experiments 1 and 2, respectively

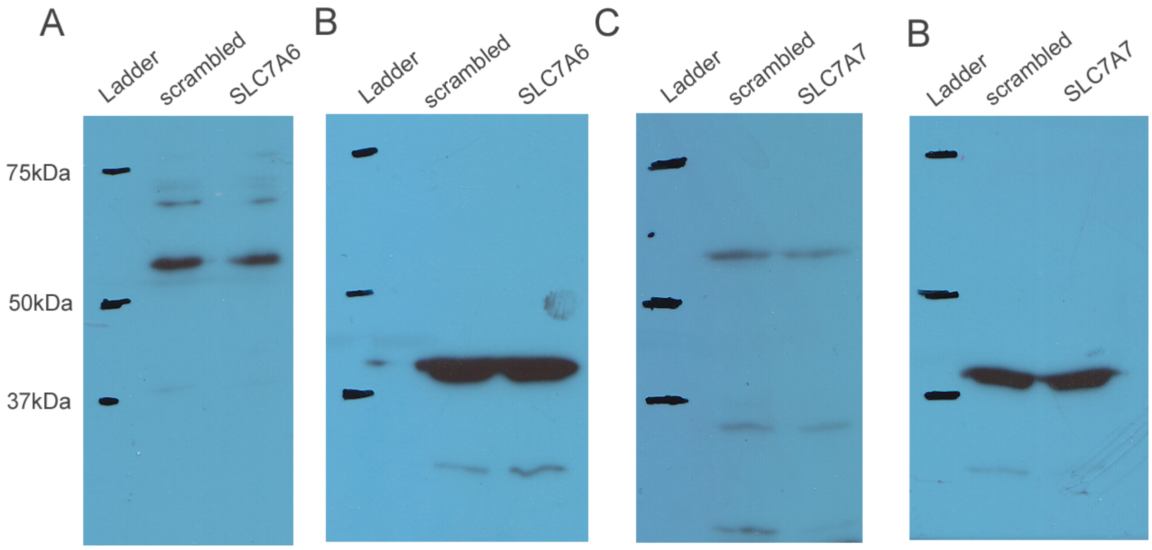

Figure S2. Blots for Figure 5C. Here above we show the original images of blots obtained for the analysis of SLC7A6/y+LAT2 and SLC7A7/y+LAT1 in A549 cells upon SLC7A6 or SLC7A7 silencing; two independent experiments were performed and one is now shown as representative.

Panel A: SLC7A6/y+LAT2, detected with a rabbit polyclonal antibody (1:500; Thermo Fisher Scientific, Italy); MW= 57 kDa. *Panel C*: SLC7A7/y+LAT1, detected with a rabbit polyclonal antibody (1:500; Thermo Fisher Scientific, Italy); MW= 57 kDa. *Panels B and D*: α-actin, detected with a polyclonal antibodies (1:1000; Sigma Aldrich, Italy), and employed as internal standard for A and C, respectively; MW= 43 kDa

Lanes:

- ✓ *Ladder* is the marker for protein dimension (same sizes in A, B, C, and D)
- ✓ *scrambled* are lysates from cells transfected with scrambled siRNA;
- ✓ *SLC7A6* and *SLC7A7* are lysates from cells transfected with SLC7A6 and SLC7A7 siRNA, respectively
